# Behavioral and health-factor domains of Life’s Essential 8 in advanced cardiovascular-kidney-metabolic syndrome among Korean adults: a nationally representative cross-sectional analysis

**DOI:** 10.3389/fendo.2026.1905093

**Published:** 2026-08-05

**Authors:** Jung Ah Lim, Kyungdo Han, Sung Hoon Yu, Jung Hwan Park, Shinje Moon, Chang Beom Lee, Sangmo Hong

**Affiliations:** 1 Department of Endocrinology and Metabolism, Hanyang University Guri Hospital, Hanyang University College of Medicine, Guri, Republic of Korea; 2 Department of Statistics and Actuarial Science, Soongsil University, Seoul, Republic of Korea; 3 Department of Endocrinology and Metabolism, Hanyang University Seoul Hospital, Hanyang University College of Medicine, Seoul, Republic of Korea

**Keywords:** behavioral domain, cardiovascular health, cardiovascular-kidney-metabolic syndrome, KNHANES, Korean adults, Life’s Essential 8, lipid-lowering medication

## Abstract

**Introduction:**

Life’s Essential 8 (LE8) summarizes cardiovascular health through four behavioral and four health-factor metrics, whereas cardiovascular-kidney-metabolic (CKM) syndrome staging characterizes disease burden and severity. Whether the two LE8 domains associate differently with advanced CKM syndrome in an Asian population remains unclear.

**Methods:**

We analyzed 17,351 Korean adults aged 20–79 years from the Korea National Health and Nutrition Examination Survey (2019–2021 and 2024) using a Korean-adapted, American Heart Association-aligned CKM framework incorporating Korean adiposity thresholds, the Korean cardiovascular risk equation, and Korean-specific LE8 body mass index scoring. Advanced CKM (Stage ≥ 3) was the primary outcome. Survey-weighted logistic regression estimated adjusted odds ratios (aORs) per 10-point LE8 increment, with prespecified sequential CKM-spectrum restriction, four outcome definitions, and medication-stratified lipid analyses.

**Results:**

The weighted prevalence of advanced CKM was 4.13% (931 cases). Each 10-point higher LE8 score was associated with lower odds of advanced CKM (aOR 0.765, 95% confidence interval [CI] 0.716–0.818). Behavioral and health-factor subscores remained independently associated after mutual adjustment. Across sequential CKM-spectrum restrictions, the behavioral association remained stable (aORs 0.879–0.897), whereas the health-factor association attenuated to non-significance at the most restricted level (aOR 0.998). Participants in the lowest LE8 quintile had 3.32-fold higher odds than those in the highest quintile (95% CI 2.30–4.80).

**Discussion:**

Higher LE8-defined cardiovascular health was inversely associated with advanced CKM; behavioral-domain associations remained stable across CKM-spectrum restriction, whereas health-factor associations require cautious interpretation owing to stage-definition overlap and treatment-related score modification. The cross-sectional design precludes causal inference.

## Introduction

The cardiovascular-kidney-metabolic (CKM) syndrome was articulated in the 2023 American Heart Association (AHA) Presidential Advisory as an integrated framework linking metabolic risk factors, chronic kidney disease, and cardiovascular disease (1–3). The CKM staging system classifies individuals from CKM Stage 0, with no CKM risk factors, to CKM Stage 4, with clinically established cardiovascular disease. Advanced CKM, commonly defined as CKM Stage ≥ 3, includes individuals with subclinical disease, very high kidney risk, high predicted cardiovascular risk, or clinical cardiovascular disease, and identifies a population that may warrant intensified risk reduction. Advanced CKM comprises two distinct components: CKM Stage 3, defined by subclinical cardiovascular disease, very-high-risk chronic kidney disease, or high predicted cardiovascular risk; and CKM Stage 4, defined by clinically established cardiovascular disease. Because Stage 3 is characterized primarily by predicted risk and subclinical markers, whereas Stage 4 reflects overt clinical disease, these stages may differ in their associations with modifiable cardiovascular health metrics. In the United States, approximately 15% of adults are in CKM Stages 3 or 4 (4). In South Korea, a serial Korea National Health and Nutrition Examination Survey (KNHANES) analysis reported a pooled advanced CKM prevalence of approximately 10% from 2011 to 2021 when CKM Stage 3 was ascertained using the AHA PREVENT equations (5).

Life’s Essential 8 (LE8) defines cardiovascular health using four behavioral metrics — diet, physical activity, nicotine exposure, and sleep — and four health-factor metrics — body mass index, blood lipids, blood glucose, and blood pressure — each scored from 0 to 100 (6). LE8 is conceptually related to CKM staging, but the two constructs are not identical. Prior studies from China and the United States suggest that behavioral LE8 metrics may show more consistent associations with advanced CKM, whereas health-factor associations may attenuate with disease progression or treatment-related risk-factor modification (7–9). In Korea, the most directly comparable analysis examined KNHANES 2013–2020 across the full CKM spectrum and reported predominantly health-factor-domain associations, with stronger associations among younger adults (10). However, Korean evidence focused specifically on prevalent advanced CKM remains limited, and few studies have examined whether behavioral and health-factor domains show consistent associations across clinically defined and risk-defined subsets of advanced CKM.

A further methodological issue concerns the interpretation of the LE8 lipid component in populations with advanced CKM. The LE8 blood-lipid metric is based on measured non-HDL cholesterol and incorporates lipid-lowering medication use in its scoring algorithm. In prevalent advanced CKM, particularly among individuals with established cardiovascular disease, lipid-lowering therapy is common and may yield favorable measured lipid values despite high underlying cardiometabolic risk. Lipid-component associations may therefore be influenced by treatment-related score modification and confounding by indication. Examining lipid-component patterns according to lipid-lowering medication use may help distinguish whether observed lipid associations reflect untreated lipid burden, treatment effects, or limitations of cross-sectional risk-factor measurement.

Korean-specific operationalization is also important because cardiometabolic risk thresholds and cardiovascular risk prediction differ across populations. Prior Korean CKM analyses have generally retained the US-derived PREVENT equation for CKM Stage 3 ascertainment and the original AHA 2022 LE8 BMI cut-points, whereas Korean guidelines define overweight and obesity at lower BMI thresholds and a Korean Cardiovascular Risk Prediction Model (K-CVD) is available (11–13). Therefore, a Korean-adapted LE8–CKM framework may provide a more clinically relevant assessment in Korean adults. To address these gaps, we evaluated the association between LE8 and advanced CKM syndrome in a contemporary, nationally representative Korean adult sample from KNHANES 2019–2021 and 2024 — survey years with complete CKM Stage 4 ascertainment — using Korean-adapted CKM and LE8 operational definitions. We further examined behavioral and health-factor domains across progressively restricted CKM-spectrum populations. Finally, based on the potential for treatment-related modification of measured lipid values, we evaluated medication-stratified lipid-component patterns as a secondary analysis.

## Materials and methods

### Study design and population

We conducted a cross-sectional analysis of KNHANES 2019–2021 and 2024, a nationally representative survey conducted by the Korea Disease Control and Prevention Agency (14), using a stratified, multistage, clustered probability sampling design.

**Figure 1:**
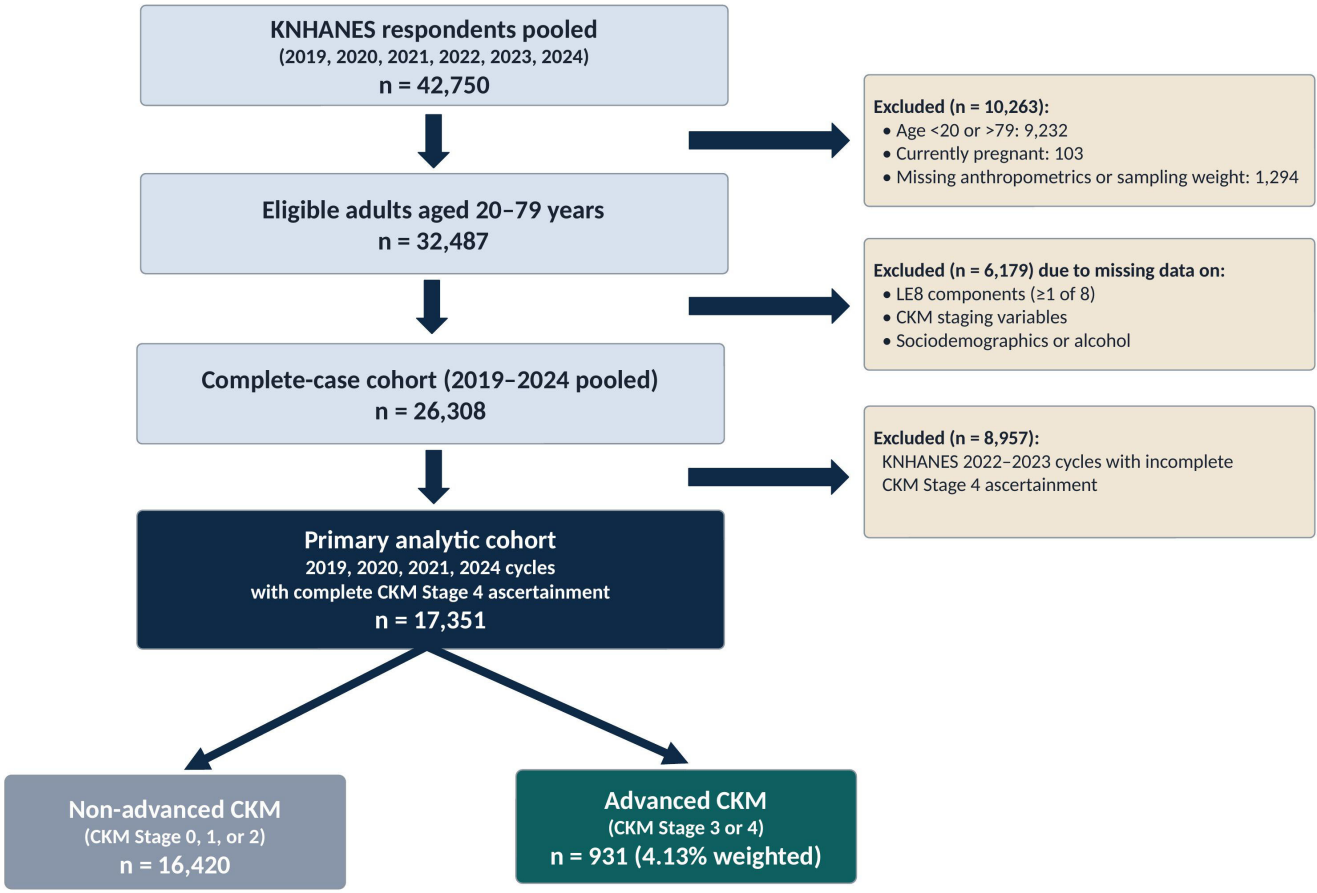
Participant flow diagram for the present analysis (Korean adults, KNHANES 2019–2024). Cohort assembly from pooled KNHANES respondents to the primary analytic cohort (n = 17,351). Reasons for exclusion at each step are shown in shaded boxes (right). The full-cohort sensitivity (n = 26,308) corresponds to the complete-case cohort across all six KNHANES cycles (2019–2024); the primary analytic cohort restricts to the four cycles (2019, 2020, 2021, 2024) with complete CKM Stage 4 ascertainment. CKM, cardiovascular-kidney-metabolic; DI3–DI6, KNHANES interview items for self-reported physician-diagnosed stroke, myocardial infarction, and angina; KNHANES, Korea National Health and Nutrition Examination Survey; LE8, Life’s Essential 8.

The primary cohort was restricted to survey years with complete CKM Stage 4 ascertainment, because physician-diagnosed stroke, myocardial infarction, and angina items were administered in 2019–2021 and 2024 but not in 2022–2023. Eligible participants were adults aged 20–79 years with complete LE8, CKM staging, covariate, and survey-design data. The final primary analytic cohort included 17,351 adults; the full sensitivity cohort included 26,308 adults. Participant flow is shown in **Figure 1**.

KNHANES participants provided written informed consent. As this study used publicly available, de-identified data, additional institutional review board approval was not required.

### Exposure: life’s essential 8 cardiovascular health score

The LE8 score was constructed per the 2022 American Heart Association Presidential Advisory (6) with KNHANES-specific adaptations. Each component was scored 0–100; the LE8 total score and the behavioral and health-factor subscores were the unweighted means of the eight, four behavioral, and four health-factor components, respectively.

The diet component used a single 24-hour dietary recall via a DASH-style six-nutrient algorithm (15, 16); DASH-style adherence has been associated with CKM staging in NHANES (17). The body mass index (BMI) component used KSSO-aligned Korean cut-points. Component-by-component scoring rules and Korean adaptations are provided in **Supplementary Table 1**.

The primary exposures were the LE8 total score, behavioral subscore, and health-factor subscore, each modeled per 10-point increment.

### Outcome: cardiovascular-kidney-metabolic syndrome staging

CKM stage was assigned hierarchically according to the AHA 2023 Cardiovascular-Kidney-Metabolic Health Presidential Advisory (1, 2). CKM Stage 1 was defined by excess adiposity or prediabetes. CKM Stage 2 was defined by AHA-aligned metabolic risk factors (18), diabetes, or moderately increased-to-high kidney risk; estimated glomerular filtration rate was computed using the CKD-EPI 2021 race-free creatinine equation (19). CKM Stage 3 was defined by very high kidney risk per the KDIGO 2012 heat map (red zone) (20) or by predicted 10-year cardiovascular disease risk ≥ 20% from the K-CVD (13). CKM Stage 4 was defined by self-reported physician-diagnosed stroke, myocardial infarction, or angina. Detailed operational criteria, including all cut-points and ascertainment variables, are provided in **Supplementary Table 2**.

The primary outcome was advanced CKM (CKM Stage 3 or 4).

### Statistical analysis

All analyses incorporated the KNHANES complex survey design using strata, primary sampling units, and pooled examination weights, with the primary cohort analyzed as a prespecified subpopulation within the 2019–2024 sampling frame. Continuous variables are presented as survey-weighted means with standard errors, and categorical variables as survey-weighted percentages. Two-sided P values were obtained from design-based, second-order Rao–Scott corrected Wald F-tests; no adjustment for multiplicity was applied.

The primary analysis evaluated the association between the LE8 total score and advanced CKM syndrome. Survey-weighted multivariable logistic regression estimated adjusted odds ratios (aORs) and 95% confidence intervals for advanced CKM per 10-point increment in the LE8 total score. Three models were fitted: Model 0 was unadjusted; Model 1 was adjusted for age and sex; and Model 2, the primary fully adjusted model, was additionally adjusted for educational attainment, household income quintile, and alcohol use.

To examine whether the overall LE8 association differed between the behavioral and health-factor domains, we performed domain-level analyses using the behavioral and health-factor subscores, modeled in two ways: each subscore entered separately into Model 2 to estimate its individual association, and both subscores entered simultaneously into the same Model 2 specification to assess whether each domain retained an independent association after mutual adjustment. Component-level analyses then entered all eight LE8 components simultaneously in Model 2, with the LE8 total score omitted.

To assess whether domain-level associations were influenced by overlap between CKM stage definitions and LE8 health-factor metrics, we performed sequential CKM-spectrum restriction analyses in three nested cohorts: L1, the full primary cohort (n = 17,351); L2, excluding CKM Stage 0 (n = 14,272); and L3, excluding CKM Stages 0 and 1 (n = 11,121). These analyses were intended to determine whether behavioral and health-factor associations remained stable after progressively removing early CKM stages, which are defined largely by adiposity, prediabetes, and metabolic risk factors.

To evaluate the influence of CKM outcome definition, we compared four prespecified outcome definitions (Outcomes A–D). These contrasts assessed whether the LE8 associations differed according to inclusion of CKM Stage 4 and according to whether CKM Stage 3 was defined narrowly by very-high-risk kidney disease or more broadly by inclusion of the K-CVD predicted-risk criterion.

Subgroup and interaction analyses evaluated heterogeneity of the primary LE8 association. Effect modification by sex, age (continuous), and diabetes status was tested using multiplicative interaction terms in Model 2, and stratum-specific estimates were calculated by sex, age category, diabetes status, and current lipid-lowering medication use. Because the LE8 lipid component may be modified by lipid-lowering therapy, medication-stratified analyses were also performed for the LE8 total score, lipid component, and health-factor subscore.

Finally, three sensitivity analyses assessed robustness of the primary findings: (i) the full pooled 2019–2024 complete-case cohort (n = 26,308); (ii) a narrow advanced CKM definition consisting of KDIGO red-zone CKM Stage 3 or CKM Stage 4 only, excluding K-CVD-based CKM Stage 3 (Outcome B); and (iii) the original AHA 2022 LE8 BMI cut-points rather than the KSSO-aligned cut-points (Pure AHA reference). Quintile-based dose-response analyses used population quintiles of the LE8 total score and subscores, with the highest quintile as the reference. Analyses used IBM SPSS Statistics version 28.0.1.0 (IBM Corp., Armonk, NY, USA) with the Complex Samples module.

## Results

### Participant characteristics

The primary analytic cohort included 17,351 adults aged 20–79 years from KNHANES 2019, 2020, 2021, and 2024 — survey years with complete CKM Stage 4 ascertainment. Advanced CKM (CKM Stage 3 or 4) was identified in 931 participants, corresponding to an unweighted proportion of 5.37% and a weighted prevalence of 4.13% after applying KNHANES survey weights with the complex sampling design. Participants with advanced CKM were substantially older than those without (weighted mean age 64.9 vs 46.9 years), more likely male (61.5% vs 49.1%), and had markedly higher prevalence of hypertension (76.7% vs 44.6%), diabetes (40.6% vs 11.8%), and current lipid-lowering medication use (43.9% vs 14.0%). Mean LE8 total score was lower in the advanced CKM group than in the non-advanced group (64.0 vs 70.9), with concordant patterns for both behavioral (67.8 vs 71.3) and health-factor (60.2 vs 70.5) subscores. Detailed participant characteristics are presented in **Table 1**. Of the 931 unweighted advanced CKM cases, 100 (10.7%) were classified as CKM Stage 3 and 831 (89.3%) as CKM Stage 4.

**Table 1:** Baseline characteristics by advanced CKM syndrome status.

Characteristic	Non-advanced (n=16,420)	Advanced CKM (n=931)
Sociodemographic characteristics		
Age, years	46.9 ± 0.2	64.9 ± 0.5
Male, %	49.1	61.5
Education, %		
≤ Elementary	9.2	30.0
Middle school	7.4	16.3
High school	36.1	29.6
≥ College	47.3	24.0
Household income quintile, %		
Q1 (lowest)	8.6	23.3
Q2	15.1	23.9
Q3	22.2	18.6
Q4	26.1	16.8
Q5 (highest)	27.9	17.3
Lifestyle factors		
Smoking status, %		
Never	61.0	44.6
Former	21.0	35.9
Current	18.0	19.5
Alcohol use, %		
Never	7.0	14.9
Former	16.0	28.4
Current	77.0	56.7
Anthropometric measures		
BMI, kg/m²	24.15 ± 0.04	24.93 ± 0.14
Waist circumference, cm	83.7 ± 0.1	89.1 ± 0.4
Blood pressure and glycemic measures		
SBP, mmHg	117.7 ± 0.2	127.7 ± 0.7
DBP, mmHg	75.6 ± 0.1	76.0 ± 0.4
HbA1c, %	5.68 ± 0.01	6.27 ± 0.04
Fasting glucose, mg/dL	100.0 ± 0.2	111.4 ± 1.1
Lipid profile		
Total cholesterol, mg/dL	193.1 ± 0.4	163.7 ± 1.8
HDL-C, mg/dL	54.3 ± 0.1	48.2 ± 0.5
LDL-C, mg/dL	114.1 ± 0.4	87.4 ± 1.6
Triglycerides, mg/dL	129.7 ± 1.1	143.2 ± 4.5
Kidney function		
eGFR, mL/min/1.73 m²	102.6 ± 0.2	82.0 ± 0.9
UACR, mg/g	14.5 ± 0.7	119.7 ± 14.0
Comorbidities, %		
Hypertension	44.6	76.7
Diabetes	11.8	40.6
Lipid-lowering medication use	14.0	43.9
Life’s Essential 8 scores (0–100)		
Behavioral subscore	71.3 ± 0.2	67.8 ± 0.6
Health-factor subscore	70.5 ± 0.2	60.2 ± 0.7
LE8 total score	70.9 ± 0.2	64.0 ± 0.4

Continuous variables are survey-weighted mean ± SE; categorical variables are survey-weighted column percentages. Sample sizes are unweighted counts.

BMI, body mass index; CKM, cardiovascular-kidney-metabolic; DBP, diastolic blood pressure; eGFR, estimated glomerular filtration rate; HbA1c, glycated hemoglobin; HDL-C/LDL-C, high-/low-density lipoprotein cholesterol; LE8, Life’s Essential 8; SBP, systolic blood pressure; SE, standard error; UACR, urine albumin-to-creatinine ratio.

### LE8–advanced CKM association and domain decomposition

In the unadjusted survey-weighted logistic regression model, each 10-point increment in the LE8 total score was associated with a crude odds ratio of 0.672 (95% confidence interval 0.639–0.706; design-based Wald F = 244.2) for advanced CKM (**Table 2**). After adjustment for age (continuous) and sex, the corresponding adjusted odds ratio (aOR) was 0.768 (95% CI 0.720–0.819; Wald F = 64.3; Model 1). The fully adjusted Model 2, additionally adjusted for educational attainment, household income quintile, and alcohol use, yielded an aOR of 0.765 (95% CI 0.716–0.818; Wald F = 62.5; P < 0.001). The estimate was substantially attenuated after adjustment for age and sex, with little additional change after further adjustment for education, income, and alcohol use.

**Table 2:** Association of LE8 scores with advanced CKM syndrome.

Exposure	Model	aOR	95% CI	Wald F	P value
Primary exposure: LE8 total score (per 10-point increment)					
LE8 total score	Model 0 (unadjusted)	0.672	0.639–0.706	244.2	<.001
LE8 total score	Model 1 (+ age, sex)	0.768	0.720–0.819	64.3	<.001
LE8 total score	Model 2 (fully adjusted)	0.765	0.716–0.818	62.5	<.001
LE8 subscores (per 10-point increment, Model 2 fully adjusted)					
Behavioral subscore	Separate models	0.879	0.840–0.920	30.9	<.001
Health-factor subscore	Separate models	0.857	0.814–0.903	33.4	<.001
Behavioral subscore	Mutually adjusted	0.887	0.847–0.929	26.1	<.001
Health-factor subscore	Mutually adjusted	0.863	0.820–0.910	30.5	<.001

Estimates from survey-weighted logistic regression (n = 17,351; 931 cases). Model 0: unadjusted. Model 1: + age, sex. Model 2 (primary): + education, income, alcohol. Exposures per 10-point increment. See Methods for cohort and CKM definitions.

Wald F: design-based test of model effects (Rao-Scott second-order). Subscores in the mutually adjusted specification share a single model. P values are two-sided. aOR, adjusted odds ratio; CI, confidence interval; LE8, Life’s Essential 8.

In single-subscore Model 2 specifications, each 10-point increment in the LE8 behavioral subscore was associated with aOR 0.879 (95% CI 0.840–0.920; Wald F = 30.9), and each 10-point increment in the LE8 health-factor subscore was associated with aOR 0.857 (95% CI 0.814–0.903; Wald F = 33.4) for advanced CKM (**Table 2**). After mutual adjustment in a single model containing both subscores simultaneously, the behavioral and health-factor estimates were largely unchanged (behavioral aOR 0.887, 95% CI 0.847–0.929; health-factor aOR 0.863, 95% CI 0.820–0.910); both associations remained statistically significant.

### Robustness across the CKM spectrum

**Figure 2:**
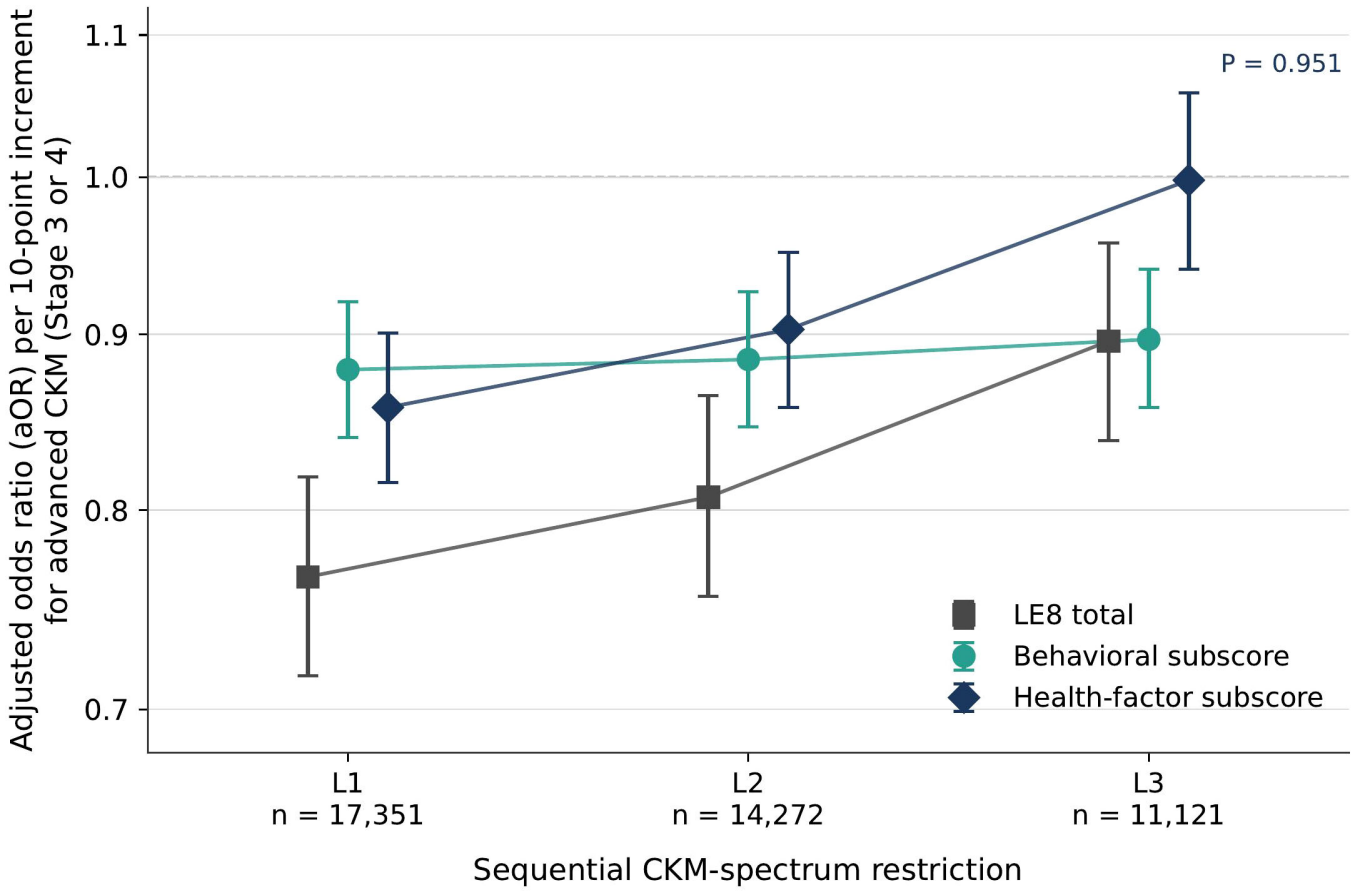
Adjusted odds ratios for advanced CKM per 10-point LE8 behavioral and health-factor subscore increment across sequentially restricted cohorts (L1, L2, and L3). Each marker shows the adjusted odds ratio (aOR) per 10-point increment in the corresponding Life’s Essential 8 (LE8) score from the primary multivariable model, estimated within three sequentially restricted cohorts: L1, full primary analytic cohort (n = 17,351; 931 cases); L2, CKM Stage 1 or higher (CKM Stage 0 excluded; n = 14,272; 931 cases); L3, CKM Stage 2 or higher (CKM Stages 0 and 1 excluded; n = 11,121; 931 cases). Horizontal bars indicate 95% confidence intervals; the dashed vertical line indicates the null reference value of 1.00. Behavioral and health-factor subscores were modeled separately in single-subscore Model 2 specifications.

When the primary association was repeated within sequentially restricted samples, the behavioral-subscore association remained stable (aORs 0.879, 0.885, 0.897 per 10-point increment at L1, L2, and L3, respectively; all P < 0.001), whereas the health-factor subscore association attenuated progressively and lost statistical significance at the most restricted level (aORs 0.857, 0.903, 0.998; P < 0.001 at L1, P < 0.001 at L2, and P = 0.951 at L3) (**Figure 2**). The LE8 total score association also moved progressively closer to the null value across restrictions (aORs 0.765, 0.807, 0.896 at L1, L2, L3) but remained statistically significant at all three levels (all P ≤ 0.003).

In a separate outcome-definition analysis (**Supplementary Table 3**), behavioral-domain estimates remained directionally consistent across the four definitions, whereas health-factor-domain estimates were more sensitive to outcome definition and became substantially stronger after excluding CKM Stage 4, particularly under Outcome C (aOR 0.606 vs 0.857 for Outcome A). The narrow-versus-expanded CKM Stage ≥ 3 contrast (Outcome A vs B) produced only marginal change in the LE8 total estimate (0.765 vs 0.786).

### Lipid-component and lipid-lowering medication analyses

**Figure 3:**
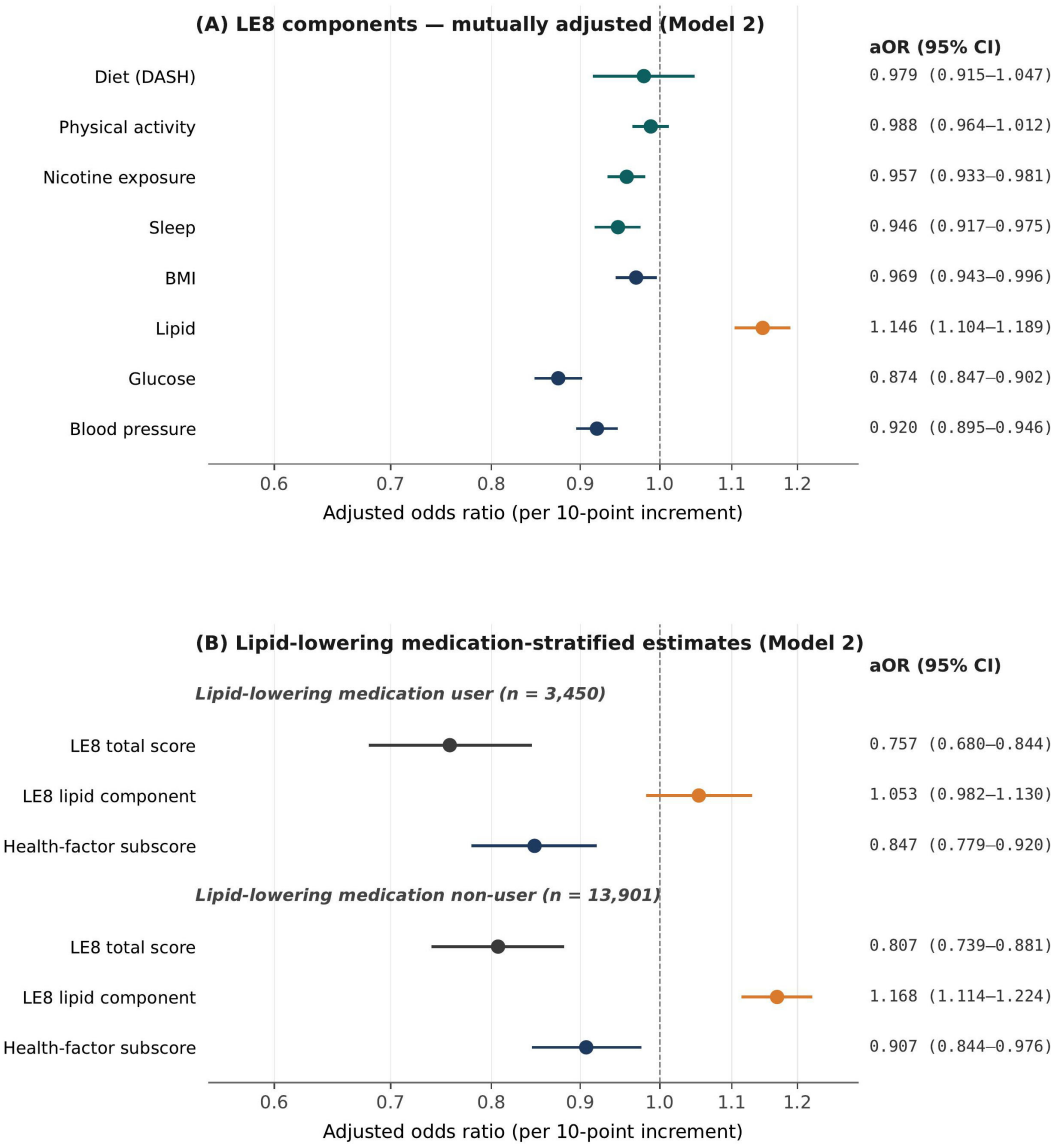
Forest plots of adjusted odds ratios (aOR) for advanced cardiovascular-kidney-metabolic (CKM) syndrome (CKM Stage 3 or 4) per 10-point increment in **(A)** the eight individual Life’s Essential 8 (LE8) components in a single mutually-adjusted Model 2 regression, and **(B)** the LE8 total score, lipid component, and health-factor subscore, stratified by current use of lipid-lowering medication, in the primary analytic cohort of 17,351 Korean adults from the Korea National Health and Nutrition Examination Survey 2019, 2020, 2021, and 2024. Survey-weighted Model 2 estimates (per 10-point LE8 increment) in the primary analytic cohort (n = 17,351; 931 advanced CKM cases). Panel A: eight LE8 components in a single mutually-adjusted regression; the lipid component is identified separately. **(B)** lipid-medication-stratified estimates (user n = 3,450, non-user n = 13,901) for LE8 total, lipid component, and health-factor subscore. Horizontal bars indicate 95% confidence intervals; the dashed vertical line indicates the null reference value of 1.00; numerical aOR (95% CI) values are shown to the right of each row. aOR, adjusted odds ratio; CI, confidence interval; CKM, cardiovascular-kidney-metabolic; KNHANES, Korea National Health and Nutrition Examination Survey; LE8, Life’s Essential 8.

Component-level analysis showed that the LE8 lipid metric was positively associated with advanced CKM, whereas the other seven LE8 components showed inverse associations (**Figure 3A**). In medication-stratified analyses, the lipid-component association differed by current lipid-lowering medication use (**Figure 3B**). Among participants currently on lipid-lowering medication, the LE8 lipid component showed no association with advanced CKM (aOR 1.053, 95% CI 0.982–1.130, P = 0.149). Among non-users, the lipid metric was positively associated with advanced CKM (aOR 1.168, 95% CI 1.114–1.224, P < 0.001). The LE8 total score and health-factor subscore associations remained inverse and significant in both medication strata [medication user: total aOR 0.757 (95% CI 0.680–0.844), health-factor aOR 0.847 (95% CI 0.779–0.920); non-user: total aOR 0.807 (95% CI 0.739–0.881), health-factor aOR 0.907 95% CI 0.844–0.976)].

### Subgroup analyses

**Figure 4:**
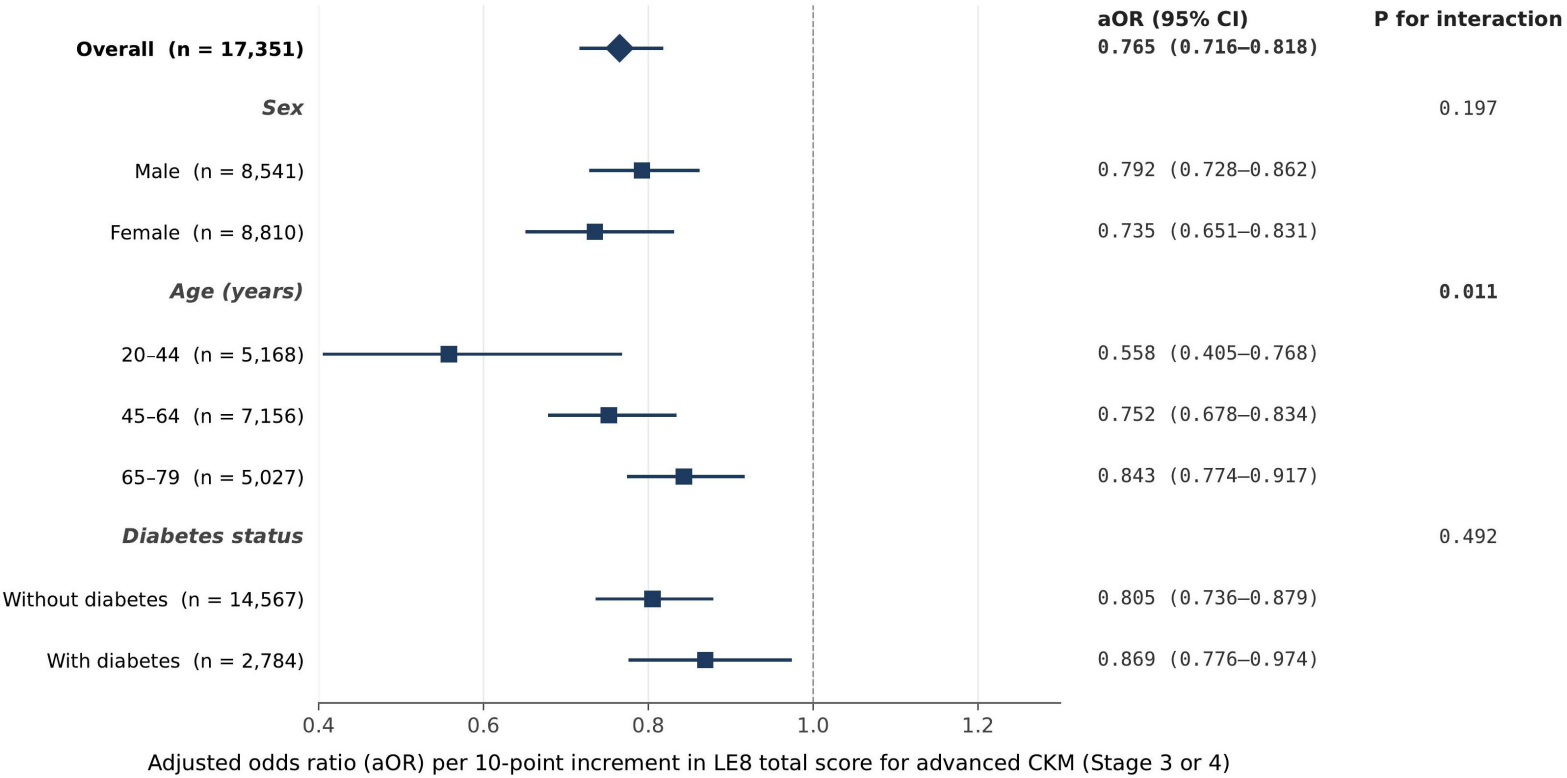
Forest plot of adjusted odds ratios (aOR) for advanced cardiovascular-kidney-metabolic (CKM) syndrome (CKM Stage 3 or 4) per 10-point increment in Life’s Essential 8 (LE8) total score, in the overall primary analytic cohort and in pre-specified subgroups defined by sex, age category, and diabetes status. Survey-weighted Model 2 estimates for LE8 total (per 10-point increment) in the primary analytic cohort (n = 17,351; 931 advanced CKM cases). Age (continuous) omitted from age-stratified models; sex from sex-stratified models. Horizontal bars: 95% CI; dashed line: null reference (aOR = 1.000); numerical aOR (95% CI) values are shown to the right of each row. P for interaction (LE8 × moderator) is shown adjacent to each stratifier subheading. aOR, adjusted odds ratio; CI, confidence interval; CKM, cardiovascular-kidney-metabolic; KNHANES, Korea National Health and Nutrition Examination Survey; LE8, Life’s Essential 8.

Effect-modification testing showed a statistically significant LE8 × age interaction (continuous age; Wald F = 6.54, P = 0.011), whereas the LE8 × sex (Wald F = 1.67, P = 0.197) and LE8 × diabetes (Wald F = 0.47, P = 0.492) interactions were not statistically significant. Stratum-specific estimates with corresponding interaction tests are presented in **Figure 4**; detailed estimates of the LE8 total score and both subscores are given in **Supplementary Table 4**.

Inverse associations of the LE8 total score with advanced CKM were observed across most strata, with stratum-level point estimates broadly consistent with the primary aOR of 0.765. Sex- and diabetes-stratified estimates were broadly consistent with the primary association; age-stratified estimates showed a gradient, with stronger associations among younger adults [aORs 0.558 (95% CI 0.405–0.768) for ages 20–44, 0.752 (0.678–0.834) for ages 45–64, and 0.843 (0.774–0.917) for ages 65–79].

### Sensitivity and dose-response analyses

Full-cohort and Pure-AHA sensitivity analyses were directionally consistent with the primary result (**Supplementary Table 5**). In the full pooled 2019–2024 cohort sensitivity analysis (n = 26,308; 988 cases; weighted prevalence 3.21%), the aOR was 0.725 (95% CI 0.681–0.772), directionally consistent with the primary estimate. The lower weighted prevalence in the full cohort was consistent with incomplete CKM Stage 4 ascertainment in 2022–2023, when cycle-specific advanced CKM prevalence was 0.6–0.7% compared with 4.8–5.9% in 2019–2021 and 2024. Substitution of the original AHA 2022 LE8 BMI cut-points for the KSSO-adapted cut-points (Pure AHA reference) yielded an aOR of 0.755 (95% CI 0.706–0.809). In the narrow advanced CKM definition, corresponding to Outcome B in the four-outcome contrasts (**Supplementary Table 3**), the aOR was 0.786 (95% CI 0.735–0.840).

Quintile-based analysis showed a graded dose-response association for the LE8 total score: compared with participants in the highest quintile (Q5; reference), participants in Q1 had 3.32-fold higher odds of advanced CKM (95% CI 2.30-4.80), with a monotonic gradient across Q4–Q2 (linear-trend per-quintile aOR 0.775 (95% CI 0.724-0.830), P < 0.001). Behavioral and health-factor subscore quintile analyses showed directionally consistent dose-response patterns (**Supplementary Table 6**).

## Discussion

In this nationally representative cross-sectional analysis of 17,351 Korean adults under a Korean-adapted, AHA-aligned CKM operationalization, higher Life’s Essential 8 (LE8)–defined cardiovascular health was substantially associated with lower advanced CKM burden (CKM Stage ≥ 3; per 10-point increment, aOR 0.765, 95% CI 0.716–0.818). Quintile-based analysis further illustrated the magnitude of this association, with participants in the lowest LE8 quintile having 3.32-fold higher odds of advanced CKM than those in the highest quintile (95% CI 2.30–4.80). Both the behavioral and health-factor subscores were independently associated with advanced CKM after mutual adjustment, indicating that the overall LE8 association was not attributable to a single domain alone. However, the two domains showed different patterns across sequential CKM-spectrum restriction: behavioral-subscore associations remained stable across L1–L3, whereas health-factor-subscore associations attenuated to non-significance at L3. These contrasting patterns suggest that the full LE8 score captures both the cumulative burden of cardiovascular health and domain-specific information that may differ in interpretability across the CKM spectrum. Thus, the overall LE8 construct remains clinically relevant, while the behavioral and health-factor domains provide complementary insights into advanced CKM assessment. Additional analyses showed stronger LE8 associations among younger adults (LE8 × age interaction P = 0.011), and a lipid-component pattern compatible with treatment-related score modification.

The behavioral-subscore robustness across our restricted CKM-spectrum subsamples is directionally consistent with prospective cohorts focused on advanced CKM (7, 8) and with broader longitudinal LE8 evidence in general populations (21–23). In 100,727 Chinese adults followed for a median of 10.1 years, Li et al. (7) reported that the population attributable fraction of suboptimal behavior was higher in CKM Stages 3–4 than in CKM Stages 0–2 (18.2% vs 13.1%), whereas the corresponding health-factor-domain attributable fractions declined across stages (51.2% to 25.2%). Tan et al. (8) in 10,321 NHANES participants with advanced CKM, and Tu et al. (9) in a complementary NHANES analysis, both identified the LE8 health behavior score as the most consistent mortality predictor (e.g., all-cause mortality HR 0.80 per 10-point behavioral-subscore increase in Tan et al.), while the health-factor score showed no significant mortality association. The convergence across distinct populations, study designs, and analytic frameworks supports that the behavioral-domain robustness in advanced CKM is not artifactual to any single methodological choice. The present analysis extends this pattern by demonstrating that the health-factor-domain association attenuates to non-significance at the most restricted CKM-spectrum level (L3), whereas the behavioral-domain association remains stable.

Within the Korean literature, Lee et al. (10) reported in 8,150 Korean adults from KNHANES 2013–2020 that the LE8–CKM association across the full CKM spectrum was driven primarily by the health-factor domain (high-versus-low health-factor weighted odds ratio 0.09; behavioral domain 0.63), with stronger associations observed among younger adults. The pronounced health-factor-domain prominence across the full spectrum reflects, in part, that early CKM stages are defined by adiposity, prediabetes, and metabolic abnormalities that overlap directly with LE8 health-factor metrics. Our findings complement this prior Korean evidence by showing that, in advanced CKM-focused analyses with sequential CKM-spectrum restriction, behavioral LE8 associations remain stable after restricting early CKM stages, whereas health-factor-domain associations are more susceptible to stage-definition overlap and to treatment-related attenuation when clinically established CVD is included. These findings are not contradictory. Health-factor-domain associations are expected to be prominent across the full CKM spectrum because early CKM stages are defined largely by adiposity, prediabetes, hypertension, dyslipidemia, and diabetes. In contrast, advanced CKM-focused analyses, particularly after sequential restriction of early CKM stages, may reveal the complementary contribution of behavioral metrics after the direct overlap between early-stage CKM definitions and LE8 health-factor metrics is reduced. We further detected a statistically significant LE8 × age interaction (P = 0.011), with stronger LE8 total associations among younger adults (aOR 0.558 in ages 20–44, 0.752 in 45–64, and 0.843 in 65–79 years), consistent with the broader age-gradient pattern reported by Lee et al. across the full CKM spectrum, and extending it to advanced CKM-focused analyses.

The relationship between LE8 and advanced CKM should be interpreted in light of partial conceptual and definitional overlap. LE8 health-factor metrics, including body mass index, blood pressure, glucose, and lipids, are closely related to the metabolic criteria used in CKM staging; therefore, associations involving the LE8 total score or the health-factor domain are not fully independent of the CKM definition. However, the comparison remains clinically meaningful because LE8 and CKM staging serve different purposes: LE8 summarizes preventive cardiovascular health on a continuous scale, whereas CKM staging characterizes the burden and severity of cardiovascular-kidney-metabolic disease. Accordingly, our analysis was intended to evaluate how favorable cardiovascular health profiles correspond to advanced CKM burden at the population level and to compare the relative contributions of the LE8 behavioral and health-factor domains, rather than to establish a causal or fully independent relationship.

The domain-specific analyses further help address this overlap concern. The behavioral domain has substantially less direct definitional overlap with the CKM staging criteria used in this study, whereas the health-factor domain contains components that closely correspond to CKM-defining metabolic abnormalities. In the sequential CKM-spectrum restriction analyses, the behavioral-domain association remained stable (aORs 0.879, 0.885, and 0.897 at L1–L3), whereas the health-factor-domain association attenuated to non-significance at L3. These findings suggest that the behavioral domain provides complementary information beyond the expected overlap between biological risk factors and CKM staging criteria.

The expanded CKM Stage 3 definition incorporates a predicted cardiovascular risk component (K-CVD ≥ 20%) that draws on blood pressure, lipid, glucose, and smoking variables overlapping with LE8 metrics. The four-outcome contrasts (**Supplementary Table 3**) showed that behavioral-domain estimates were relatively stable across outcome definitions, whereas health-factor-domain estimates changed more substantially when CKM Stage 4 was excluded (health-factor aOR 0.857 in Outcome A vs 0.606 in Outcome C). This pattern is consistent with dilution of health-factor-domain associations when participants with clinical CVD are included, plausibly because post-event treatment, risk-factor control, and post-diagnosis lifestyle change can improve measured blood pressure, lipid, and glycemic scores despite high underlying risk — a form of reverse causation specific to health-factor-domain metrics in cross-sectional prevalent CKM analyses; this is supported by our medication-stratified analysis (see below). The narrow-versus-expanded CKM Stage 3 contrast (Outcome A vs B) attenuated the LE8 total association only marginally (0.765 vs 0.786), indicating that the principal LE8–advanced CKM association is not primarily driven by the K-CVD component. Sensitivity analyses supported the primary finding, with persistent inverse associations in both the full pooled 2019–2024 cohort and the Pure AHA reference (**Supplementary Table 5**).

The lipid-component finding should be interpreted cautiously. This pattern may reflect treatment-related reclassification arising from the specific structure of the LE8 lipid component. The LE8 lipid metric is largely determined by measured non-HDL cholesterol, which can be directly lowered by statins or other lipid-lowering medications. Individuals with higher underlying cardiometabolic risk who receive lipid-lowering therapy may therefore be classified into a more favorable lipid-score category despite having a persistently high baseline risk. This may produce an apparent positive association because of confounding by indication or reverse causality. This treatment-related pattern may not operate identically across the blood-pressure and glucose components. The glucose component continues to reflect diabetes status and glycemic burden, whereas blood-pressure classification may be influenced by medication use, residual elevation, adherence, lifestyle factors, and measurement variability. In contrast, lipid-lowering therapy directly modifies the biomarker used in the LE8 lipid score, making the lipid component particularly susceptible to treatment-related reclassification of underlying risk. Even among non-users, however, the positive association likely reflects the limitations of cross-sectional measurement, including unmeasured cumulative lipid exposure, prior or intermittent treatment, selective survival, and disease-related reverse epidemiologic patterns, rather than a direct adverse effect of favorable lipid levels. Importantly, the LE8 total score and health-factor subscore remained inversely associated with advanced CKM in both medication strata (total aOR 0.757 among users and 0.807 among non-users; health-factor aOR 0.847 and 0.907, respectively), indicating that the principal LE8–advanced CKM association was not contingent on the lipid component.

A principal strength of this study is the integrated application of Korean-adapted operational components — KSSO 2018 BMI and waist circumference cut-points for CKM Stage 1, the K-CVD equation for CKM Stage 3 cardiovascular risk (13), and KSSO-aligned LE8 BMI scoring (12) — within an AHA 2023 framework. Prior Korean CKM analyses generally retained the US-derived PREVENT equation and the AHA original LE8 BMI cut-points; few prior Korean analyses have integrated these population-specific components within a single LE8–CKM framework, and the principal behavioral-versus-health-factor finding was robust to this alignment [Pure AHA sensitivity: aOR 0.755 (0.706–0.809) vs primary 0.765 (0.716–0.818)]. A second strength is the prespecified restriction of the primary cohort to survey years with complete CKM Stage 4 ascertainment (2019, 2020, 2021, 2024), avoiding the systematic outcome underascertainment that affects 2022–2023 under the KNHANES topic-rotation schedule; the directionally consistent full-cohort sensitivity (aOR 0.725) supports robustness across cohort definitions. Additional strengths include the nationally representative sample with rigorous complex-sampling implementation, a contemporary survey period, a documented analytic pipeline using publicly available KNHANES data, separate and joint evaluation of behavioral and health-factor domains, and a set of prespecified robustness analyses (sequential CKM-spectrum restriction, four-outcome contrasts, multiple stratified analyses, mutual adjustment, and quintile dose-response).

This study has several limitations. First, the cross-sectional design precludes causal inference; the four-outcome contrasts and medication-stratified analyses indicated patterns compatible with reverse causation and treatment-related score modification rather than direct adverse effects of favorable LE8 lipid scores, although residual mathematical coupling between LE8 health-factor metrics and CKM staging criteria cannot be fully excluded. Second, CKM Stage 4 was ascertained from self-reported physician-diagnosed stroke, myocardial infarction, or angina; KNHANES does not uniformly capture other AHA-specified clinical CVD subtypes (e.g., heart failure, peripheral arterial disease, atrial fibrillation), and prevalent disease ascertainment is subject to survival and recall biases. Because CKM Stage 4 accounted for most advanced CKM cases in the primary cohort (831 of 931, 89.3%), the primary outcome may predominantly reflect prevalent, self-reported clinical cardiovascular disease rather than the full spectrum of advanced CKM progression. Accordingly, the findings may not apply equally to the risk-defined CKM Stage 3 phenotype or to subclinical phases of advanced CKM. To assess this influence, we compared four outcome definitions (**Supplementary Table 3**), including analyses excluding Stage 4, which yielded directionally consistent estimates for the behavioral domains. Future longitudinal studies using adjudicated cardiovascular outcomes and sufficient numbers of CKM Stage 3 cases are needed to determine whether the observed LE8-domain associations are consistent across the broader advanced CKM spectrum. Third, this analysis did not include a formal AHA PREVENT-based (24) CKM Stage 3 sensitivity, which would enable direct comparison with prior Korean CKM analyses (5, 10); this represents a planned future extension. Fourth, the LE8 diet metric was constructed from a single 24-hour recall using a DASH-style algorithm; a Korean Healthy Eating Index (KHEI)-based sensitivity was not feasible owing to the 2022 KHEI revision (25). Fifth, alcohol use was categorized at three levels without finer gradation. Finally, these findings are most directly generalizable to community-dwelling Korean adults aged 20–79 years; directional concordance with Chinese (7) and US (8, 9) prospective cohorts suggests the principal association is unlikely to be entirely Korea-specific, but prospective confirmation is needed.

## Conclusions

In this nationally representative Korean sample analyzed under a Korean-adapted, AHA-aligned CKM operationalization, higher LE8-defined cardiovascular health was associated with lower advanced CKM burden in a graded manner. In this cross-sectional sample, however, advanced CKM was predominantly represented by self-reported prevalent clinical cardiovascular disease. Therefore, the results should be interpreted as evidence of population-level association rather than causal or prognostic relevance. The overall pattern supports the relevance of the full LE8 construct, including both behavioral and health-factor components, in CKM-spectrum assessment. Behavioral-domain associations remained stable across CKM-spectrum restriction and may provide complementary information in advanced CKM assessment, whereas health-factor-domain associations require careful interpretation in cross-sectional analyses because of stage-definition overlap, post-event treatment, and treatment-related score modification. Prospective Korean studies with incident endpoints are needed to determine whether favorable LE8 trajectories are associated with lower CKM progression and clinical events.

## Data Availability

Publicly available datasets were analyzed in this study. This data can be found here: https://knhanes.kdca.go.kr.
