# Supplementary material for: Behavioral and health-factor domains of Life’s Essential 8 in advanced cardiovascular-kidney-metabolic syndrome among Korean adults: a nationally representative cross-sectional analysis
Source: Front Endocrinol (Lausanne). 2026 Aug 5;17:1905093. doi: 10.3389/fendo.2026.1905093 (PMC13485572; doi:10.3389/fendo.2026.1905093)
Supplement: Supplementary file 1 [file Table1.docx]

**Supplementary Table S1.** Operational scoring of the eight Life’s Essential 8 (LE8) components with KNHANES variables and Korean adaptations.

Each LE8 component was scored on a 0–100 scale per the 2022 AHA Presidential Advisory [6]. The LE8 total score and the behavioral and health-factor subscores were the unweighted means of the eight, four behavioral, and four health-factor components, respectively. Diet was scored using a DASH-style six-nutrient algorithm [15, 16] adapted from the single 24-hour dietary recall with rank-based rescaling to a 0–100 score [17]. The body mass index (BMI) component was scored using Korean Society for the Study of Obesity (KSSO) 2018 cut-points [11] in the primary analysis; the original AHA 2022 BMI cut-points were retained for the Pure-AHA sensitivity analysis.

*Abbreviations:* AHA, American Heart Association; BMI, body mass index; CVH, cardiovascular health; DASH, Dietary Approaches to Stop Hypertension; HbA1c, glycated hemoglobin; HDL, high-density lipoprotein; KNHANES, Korea National Health and Nutrition Examination Survey; KSSO, Korean Society for the Study of Obesity; LE8, Life’s Essential 8; MET, metabolic equivalent; MVPA, moderate-to-vigorous physical activity.

| Component | KNHANES source | Scoring |
| --- | --- | --- |
| Diet | 24-h dietary recall (NUTR_24R): protein, total fiber, potassium, calcium, total fat, sodium | Each of 6 nutrients ranked into population quintiles (1–5); ranks for total fat and sodium reversed. Sum of 6 ranks (range 6–30) rescaled linearly to 0–100. |
| Physical activity | Self-reported physical activity: leisure-time vigorous (BE3_76–78) and moderate (BE3_86–88), plus walking (BE3_31–33). MVPA min/wk = 2 × vigorous + moderate + walking. | 100: ≥150 min/wk • 90: 120–149 • 80: 90–119 • 60: 60–89 • 40: 30–59 • 20: 1–29 • 0: none. |
| Nicotine exposure | Smoking history (BS variables); current and former cigarette/e-cigarette use; secondhand-smoke exposure at home or workplace | 100: never (no secondhand) • 75: former, quit ≥5 y • 50: former, quit 1–<5 y • 25: former, quit <1 y, or current cigarette only • 0: any current tobacco/e-cigarette. Subtract 20 if current secondhand exposure. |
| Sleep duration | Self-reported sleep hours per night; 7-day weighted average (weekday × 5 + weekend × 2)/7. Direct hours (BP16_1, BP16_2) for cycles 2019/2020/2022/2023; derived from reported bed and wake times (BP16_11–14, BP16_21–24) for cycles 2021/2024. | 100: 7–<9 h • 90: 9–<10 h • 70: 6–<7 h • 40: 5–<6 h or ≥10 h • 20: 4–<5 h • 0: <4 h. |
| Body mass index (KSSO; primary) | Measured height/weight (HE_ht, HE_wt) | 100: 18.5–22.9 kg/m² (KSSO normal) • 70: 23.0–24.9 (KSSO overweight) • 30: 25.0–29.9 (KSSO obesity I) • 15: 30.0–34.9 (KSSO obesity II) • 0: <18.5 or ≥35.0. |
| Body mass index (AHA; sensitivity) | Same source | 100: 18.5–<25 • 70: 25–<30 • 30: 30–<35 • 15: 35–<40 • 0: <18.5 or ≥40. |
| Blood lipids (non-HDL cholesterol) | Non-HDL = total cholesterol − HDL (HE_TC, HE_HDL_st2); current lipid-lowering medication (DI2_pt) | 100: <130 mg/dL • 60: 130–159 • 40: 160–189 • 20: 190–219 • 0: ≥220. Subtract 20 if currently on lipid-lowering medication. |
| Blood glucose (HbA1c) | HbA1c (HE_HbA1c); fasting plasma glucose (HE_glu); diagnosed diabetes (DE1_dg, DE1_pt) | Without diabetes — 100: HbA1c <5.7% • 60: 5.7–<6.5%. With diabetes — 40: HbA1c <7.0% • 30: 7.0–<8.0% • 20: 8.0–<9.0% • 10: 9.0–<10.0% • 0: ≥10.0%. |
| Blood pressure | Average of 2nd and 3rd mercury sphygmomanometer readings (HE_sbp1–3, HE_dbp1–3); current antihypertensive use (DI1_pt) | 100: <120/<80 (untreated) • 75: 120–129/<80 • 50: 130–139/80–89 • 25: 140–159/90–99 • 0: ≥160/≥100. Subtract 20 if currently on antihypertensive medication. |

**Supplementary Table S2.** Operational definitions of Cardiovascular-Kidney-Metabolic (CKM) syndrome stages with KNHANES variables and cut-points.

Stages were assigned hierarchically per the AHA 2023 CKM Presidential Advisory [1, 2], with the highest applicable stage retained. Estimated glomerular filtration rate (eGFR) was calculated from serum creatinine using the CKD-EPI 2021 race-free equation [19]. Urinary albumin-to-creatinine ratio (UACR, mg/g) = urinary albumin (mg/L) × 100 / urinary creatinine (mg/dL). KDIGO 2012 risk categories follow the heat-map in which red zone denotes very-high risk; yellow and orange zones denote moderately-increased to high risk. CKM Stage 3 by the Korean cardiovascular risk equation (K-CVD) [13] is defined as predicted 10-year CVD risk ≥ 20%. CKM Stage 4 was ascertained from KNHANES health-interview items DI3 (stroke), DI4–DI5 (myocardial infarction), and DI6 (angina); these items were administered in 2019–2021 and 2024 but not in 2022–2023, which determined the primary cohort restriction. KNHANES does not uniformly ascertain heart failure, peripheral arterial disease, or atrial fibrillation, so CKM Stage 4 is limited to physician-diagnosed stroke and coronary heart disease.

*Abbreviations:* AHA, American Heart Association; BMI, body mass index; CKD-EPI, Chronic Kidney Disease Epidemiology Collaboration; CKM, cardiovascular-kidney-metabolic; CVD, cardiovascular disease; eGFR, estimated glomerular filtration rate; HDL, high-density lipoprotein; HbA1c, glycated hemoglobin; K-CVD, Korean Cardiovascular Risk Prediction Model; KDIGO, Kidney Disease: Improving Global Outcomes; KNHANES, Korea National Health and Nutrition Examination Survey; KSSO, Korean Society for the Study of Obesity; UACR, urinary albumin-to-creatinine ratio; WC, waist circumference.

| Stage | Operational definition (any criterion satisfies the stage) |
| --- | --- |
| CKM Stage 0 | No CKM risk factors meeting CKM Stage 1–4 criteria below. |
| CKM Stage 1 (excess adiposity or prediabetes) | BMI ≥ 23 kg/m² (KSSO 2018 overweight); or WC ≥ 90 cm in men or ≥ 85 cm in women (KSSO 2018 abdominal obesity) [11]; or fasting plasma glucose 100–125 mg/dL or HbA1c 5.7–6.4% in the absence of diagnosed diabetes (prediabetes). |
| CKM Stage 2 (metabolic risk factors, diabetes, or moderately increased-to-high kidney risk) | Hypertension (SBP ≥ 130 mmHg, DBP ≥ 80 mmHg, self-reported physician diagnosis, or current antihypertensive use); or hypertriglyceridemia (triglycerides ≥ 135 mg/dL); or metabolic syndrome (NCEP-ATP III with KSSO Korean WC threshold; ≥ 3 of: WC ≥ 90 cm M or ≥ 85 cm F, triglycerides ≥ 150 mg/dL, HDL < 40 mg/dL M or < 50 mg/dL F, BP ≥ 130/80 mmHg or current antihypertensive, fasting plasma glucose ≥ 100 mg/dL or current antidiabetic); or diabetes (fasting plasma glucose ≥ 126 mg/dL, self-reported physician diagnosis, or current antidiabetic medication); or KDIGO 2012 yellow- or orange-zone kidney risk (moderately-increased to high). |
| CKM Stage 3 (very-high kidney risk or high predicted CVD risk) | KDIGO 2012 red zone: G3aA3, G3bA2, G3bA3, G4 with any albuminuria, or G5 with any albuminuria; or K-CVD-predicted 10-year CVD risk ≥ 20% (Korean Cardiovascular Risk Prediction Model, validated in 325,934 Korean adults aged 20–80 years) [13]. |
| CKM Stage 4 (clinical CVD) | Self-reported physician-diagnosed stroke (DI3); or myocardial infarction (DI4 / DI5); or angina (DI6). |

**Supplementary Table S3.** Adjusted odds ratios (aOR) for advanced cardiovascular-kidney-metabolic (CKM) syndrome per 10-point increment in Life’s Essential 8 (LE8) total score, behavioral subscore, and health-factor subscore, under four alternative outcome definitions (within the primary analytic cohort).

Survey-weighted Model 2 regression (per 10-point LE8 increment) under four alternative outcome definitions.

Outcomes: A (primary), expanded CKM Stage ≥ 3 = KDIGO red zone or K-CVD ≥ 20% or CKM Stage 4; B, narrow CKM Stage ≥ 3 (KDIGO red zone or CKM Stage 4 only, excluding K-CVD-based CKM Stage 3); C, expanded CKM Stage 3 only with CKM Stage 4 excluded; D, narrow CKM Stage 3 only (KDIGO red zone) with CKM Stage 4 excluded.

*Abbreviations:* aOR, adjusted odds ratio; CI, confidence interval; CKM, cardiovascular-kidney-metabolic; KDIGO, Kidney Disease: Improving Global Outcomes; KNHANES, Korea National Health and Nutrition Examination Survey; K-CVD, Korean Cardiovascular Risk Prediction Model; LE8, Life’s Essential 8.

| Exposure (per 10-pt ↑) | Outcome A: expanded ≥ 3 (n = 17,351; 931 events) | Outcome B: narrow ≥ 3 | Outcome C: CKM Stage 3 expanded (no CKM Stage 4) | Outcome D: CKM Stage 3 narrow KDIGO (no CKM Stage 4) |
| --- | --- | --- | --- | --- |
| LE8 total score | 0.765 (0.716–0.818) | 0.786 (0.735–0.840) | 0.478 (0.390–0.586) | 0.555 (0.448–0.688) |
| LE8 behavioral subscore | 0.879 (0.840–0.920) | 0.893 (0.852–0.936) | 0.741 (0.654–0.841) | 0.810 (0.701–0.937) |
| LE8 health-factor subscore | 0.857 (0.814–0.903) | 0.869 (0.824–0.916) | 0.606 (0.504–0.729) | 0.649 (0.535–0.788) |

**Supplementary Table S4.** Adjusted odds ratios (aOR) for advanced cardiovascular-kidney-metabolic (CKM) syndrome (CKM Stage 3 or 4) per 10-point increment in Life’s Essential 8 (LE8) total score, behavioral subscore, and health-factor subscore, stratified by sex, age category, and diabetes status (within the primary analytic cohort).

Stratum-specific Model 2 estimates (per 10-point LE8 increment). Within-stratum filtering preserves the survey design. Age (continuous) is omitted from age-stratified models; sex from sex-stratified models. Diabetes: fasting glucose ≥ 126 mg/dL, physician diagnosis, or antidiabetic medication.

* 95% confidence interval includes the null reference value of 1.000.

*Abbreviations:* aOR, adjusted odds ratio; CI, confidence interval; CKM, cardiovascular-kidney-metabolic; LE8, Life’s Essential 8.

| Subgroup | LE8 total aOR (95% CI) | LE8 behavioral aOR (95% CI) | LE8 health-factor aOR (95% CI) |
| --- | --- | --- | --- |
| *Sex* |  |  |  |
| Male (n = 8,541) | 0.792 (0.728–0.862) | 0.870 (0.821–0.921) | 0.899 (0.840–0.963) |
| Female (n = 8,810) | 0.735 (0.651–0.831) | 0.911 (0.833–0.995) | 0.807 (0.739–0.881) |
| *Age (years)* |  |  |  |
| 20–44 | 0.558 (0.405–0.768) | 0.775 (0.604–0.993) | 0.697 (0.552–0.881) |
| 45–64 | 0.752 (0.678–0.834) | 0.924 (0.856–0.997) | 0.808 (0.744–0.877) |
| 65–79 | 0.843 (0.774–0.917) | 0.896 (0.847–0.949) | 0.935 (0.874–0.999) |
| *Diabetes status* |  |  |  |
| Without diabetes | 0.805 (0.736–0.879) | 0.854 (0.806–0.904) | 0.931 (0.866–1.002)* |
| With diabetes | 0.869 (0.776–0.974) | 0.942 (0.870–1.019)* | 0.915 (0.840–0.995) |

**Supplementary Table S5.** Sensitivity analyses for the association between Life’s Essential 8 (LE8) score and advanced cardiovascular-kidney-metabolic (CKM) syndrome.

Survey-weighted Model 2 estimates (per 10-point LE8 increment).

Full-cohort sensitivity: full pooled 2019–2024 cohort (n = 26,308); CKM Stage 4 ascertainment was incomplete in 2022–2023 per KNHANES topic-rotation schedule. Pure-AHA sensitivity: AHA 2022 LE8 BMI cut-points (25/30/35/40 kg/m²) substituted for KSSO 2018 cut-points within the primary analytic cohort; behavioral subscore is unaffected and not shown.

P values are from second-order Rao-Scott corrected design-based Wald F-tests of the LE8 effect; values are two-sided.

*Abbreviations:* aOR, adjusted odds ratio; AHA, American Heart Association; BMI, body mass index; CI, confidence interval; CKM, cardiovascular-kidney-metabolic; KNHANES, Korea National Health and Nutrition Examination Survey; KSSO, Korean Society for the Study of Obesity; LE8, Life’s Essential 8.

| Sensitivity analysis | n | aOR | 95% CI | Wald F | P value |
| --- | --- | --- | --- | --- | --- |
| *Full-cohort sensitivity (CKM Stage 4 incomplete in 2022–2023)* | | | | | |
| LE8 total score | 26,308 (988 events) | 0.725 | 0.681–0.772 | 101.4 | <.001 |
| LE8 behavioral subscore | 26,308 (988 events) | 0.870 | 0.833–0.908 | 40.9 | <.001 |
| LE8 health-factor subscore | 26,308 (988 events) | 0.820 | 0.780–0.862 | 61.6 | <.001 |
| *Pure-AHA sensitivity (LE8 BMI scoring under AHA 2022 cut-points 25/30/35/40 kg/m²)* | | | | | |
| LE8 total score (AHA BMI) | 17,351 (931 events) | 0.755 | 0.706–0.809 | 64.8 | <.001 |
| LE8 health-factor subscore (AHA BMI) | 17,351 (931 events) | 0.838 | 0.795–0.883 | 43.4 | <.001 |

**Supplementary Table S6.** Dose-response analysis by Life’s Essential 8 (LE8) quintile for the association with advanced cardiovascular-kidney-metabolic (CKM) syndrome (within the primary analytic cohort).

Dose-response across LE8 score quintiles (Q5 = highest CVH, reference) within the primary analytic cohort.

Survey-weighted Model 2 regression. Panel A: quintile estimates from a single categorical model (Q5 reference); trend P from a separate ordinal-coded model (1–5).

Trend P values are from second-order Rao-Scott corrected design-based Wald F-tests of the linear-trend coefficient; values are two-sided.

* 95% confidence interval includes the null reference value of 1.000.

*Abbreviations:* aOR, adjusted odds ratio; CI, confidence interval; CKM, cardiovascular-kidney-metabolic; CVH, cardiovascular health; KNHANES, Korea National Health and Nutrition Examination Survey; LE8, Life’s Essential 8; Q, quintile.

| Exposure | Q1 vs Q5 | Q2 vs Q5 | Q3 vs Q5 | Q4 vs Q5 | Trend per-Q aOR (95% CI) | Trend P |
| --- | --- | --- | --- | --- | --- | --- |
| LE8 total score | 3.319 (2.297–4.799) | 2.245 (1.581–3.189) | 2.059 (1.419–2.987) | 1.654 (1.137–2.405) | 0.775 (0.724–0.830) | <.001 |
| LE8 behavioral subscore | 1.957 (1.503–2.551) | 1.817 (1.405–2.348) | 1.600 (1.214–2.109) | 1.210 (0.902–1.624)* | 0.844 (0.798–0.893) | <.001 |
| LE8 health-factor subscore | 2.301 (1.538–3.448) | 1.871 (1.257–2.787) | 1.684 (1.105–2.566) | 1.406 (0.932–2.123)* | 0.837 (0.781–0.897) | <.001 |
